# Prevalence of Latent Tuberculosis Infection in Vulnerable Groups in the State of Santa Catarina, Brazil

**DOI:** 10.1590/0037-8682-0329-2025

**Published:** 2026-03-27

**Authors:** Emanuela da Rocha Carvalho, Emil Kupek

**Affiliations:** 1 Universidade Federal de Santa Catarina, Departamento de Pediatria, Florianópolis, SC, Brasil.; 2 Universidade Federal de Santa Catarina, Programa de Pós-Graduação em Ciências Médicas, Florianópolis, SC, Brasil.; 3 Universidade Federal de Santa Catarina, Faculdade de Saúde Pública, Florianópolis, SC, Brasil.

**Keywords:** Latent tuberculosis, Prevalence, Risk groups, Statistics

## Abstract

**Background:**

Tuberculosis remains a major public health challenge that exerts substantial pressure on healthcare systems worldwide. Despite advances in diagnosis and treatment, the proper management of latent tuberculosis infection (LTBI) remains inefficient, especially among vulnerable populations. In the State of Santa Catarina, Brazil, the absence of comprehensive and specific records on LTBI prevalence in these at-risk groups limits effective public health planning and interventions.

**Methods:**

Interferon-gamma release assay (IGRA) test records in the State of Santa Catarina, Brazil, contained partial information on risk factors for LTBI. Multiple imputations were used to estimate the likely risk groups among individuals without such information. The study was approved by the Human Research Ethics Committee under protocol number CAAE 79499224.4.0000.5361.

**Results:**

The estimated LTBI prevalence among individuals with available IGRA results was 6.89%, with higher rates among people living with human immunodeficiency virus (HIV)/acquired immunodeficiency syndrome (18.25%) and immigrants (3.65%). Underreporting was notable among individuals deprived of liberty, those experiencing homelessness, those who use illicit drugs, and those with diabetes mellitus. Missing data were frequent among individuals living with HIV; people of African, Indigenous, or Asian descent; and those exposed to drug-resistant TB. More than 10% of LTBI cases occurred in children and adolescents aged <15 years.

**Conclusion:**

HIV was the most prevalent risk factor among IGRA-positive individuals and was substantially underestimated. An urgent expansion of LTBI identification efforts is needed for all vulnerable populations, with special attention paid to children and adolescents who present a high risk of progressing to active TB.

## INTRODUCTION

Tuberculosis continues to challenge healthcare systems and remains a major global public health issue. In Brazil, 84,308 new cases were reported in 2024, corresponding to a notification rate of 39.7 cases per 100,000 population^1^. Santa Catarina is a Brazilian state located in the southern region, with an estimated population of 7,610,361 inhabitants in 2022^2^, and reported a tuberculosis incidence rate of 26.4 cases and a related mortality rate of 1.6 per 100,000 population^1^.

Latent tuberculosis infection (LTBI) is defined as a persistent immune response to *Mycobacterium tuberculosis* antigens without clinical evidence of active disease. The lack of a gold standard test hampers accurate estimation of the global burden^3^. Individual health conditions and habits, as well as socio-environmental factors, can influence the risk of TB. For example, people living with human immunodeficiency virus (HIV)/acquired immunodeficiency syndrome (AIDS) (PLWHA) have 4-fold and 3.3-fold higher risks of active tuberculosis and LTBI, respectively^4^, compared to those without HIV infection.

Social vulnerability is associated with an increased risk of developing LTBI, with populations experiencing homelessness showing considerably higher incidence and worse outcomes in high-burden countries^5^. In some Brazilian states, tuberculosis/HIV co-infection among individuals experiencing homelessness has reached 30%^5^, whereas incarcerated individuals have been associated with LTBI rates of up to 73% in southeastern Brazil^6^. The global migration crisis, driven by conflict, climate change, and food insecurity, has increased tuberculosis vulnerability among immigrants, with tuberculosis prevalence among refugee youth estimated at 15%^7^. Children exposed to smear-positive tuberculosis, especially those under 5 years of age and adolescents, face a higher risk of progression from LTBI to active tuberculosis. Among infants under 12 months of age without preventive tuberculosis treatment, this risk increases to 40‒50%^8^. 

Despite universal BCG vaccination to protect against severe forms of tuberculosis in childhood, Brazil continues to have a high tuberculosis burden. Vaccine coverage was estimated at 84.3% in 2023, raising concerns about national public health^1^.

Active and passive tobacco exposures are associated with tuberculosis and related mortality^9^. Furthermore, diabetes mellitus is a risk factor for disease progression^10^.

Although risk factors for LTBI have been well established, its prevalence among vulnerable groups remains uncertain owing to incomplete records. Ideally, group-specific case counts and population denominators should be available; however, these are often lacking and must be estimated. Accordingly, we aimed to address this gap by evaluating secondary data from state-level interferon-gamma release assay (IGRA) laboratory records.

## METHODS

### Data sources

This study used secondary data from the Notifiable Diseases Information System (SINAN), the State Notification System for Latent Tuberculosis Infection (VIGILANTOS), the Central Public Health Laboratory of Santa Catarina database, and the Mortality Information System (SIM).

Estimates of LTBI risk group sizes in 2023 were calculated assuming no overlap between the groups. The estimates were derived from the following data sources: (a) SINAN for PLWHA in 2023^11^; (b) the report of the State Secretariat for Prison and Socio-Educational Administration of Santa Catarina^12^ for incarcerated individuals; (c) the federal registry of the social support recipients, known as “Cadastro Único” in Portuguese, for the immigrant population in December 2023^13^; (d) the State Department for Social Assistance and non-governmental organization Observa DH (Santa Catarina)^14^ for people experiencing homelessness; e) VIGILANTOS records for individuals under 15 years of age^15^; and (f) the Third National Survey on Drug Use by the Brazilian Population (2017)^16^ for people who used crack and/or similar drugs. The prevalence of users aged 12-65 years in Brazilian capitals was estimated using the indirect method known as the *Network Scale-up Method* ^17^, resulting in approximately 380,000 users in 2015^16^. Additional data sources included (g) the Surveillance of Risk and Protective Factors for Chronic Diseases by Telephone Survey (VIGITEL) for smoking, defined as consumption of more than 20 cigarettes/day^18^; and (h) the Hospital Information System (SIH), including the total number of hospital admission authorizations (AIH) for diabetes mellitus and tuberculosis in 2023 in Santa Catarina^19^ for diabetes mellitus.

### Statistical methods

Multiple imputation (MI) is a statistical method used to address missing data by estimating values based on patterns observed in the observed dataset^20^. We assumed that the relationships between the reported risk categories and demographic and clinical characteristics observed among IGRA- or purified protein derivative (PPD)-tested individuals also apply to those with missing data. These estimates represent conditional probabilities derived from observed patterns under the assumption of missing at random^20^. For mutually exclusive risk categories recorded on the IGRA laboratory form or PPD in the VIGILANTOS System, the probability of belonging to each category was modeled using a multinomial distribution, with the following categories: HIV, illicit drug use, incarcerated population, homelessness, and immigrants. 

To account for sampling variability, 30 independent estimates were generated through repeated sampling of the available data. The mean and variance of these estimates were used to derive 95% confidence intervals (CI). These estimates were subsequently applied to calculate the probability of belonging to each risk category for records with missing information by converting the logit scale values (xb) to a probability scale (P), as described below:

$$P=e^{\left(a+xb\right)}/\left[1+e^{\left(a+xb\right)}\right]$$

where *e* is the base of the natural logarithm, *a* is the intercept of the multinomial regression on the logit scale, *x* is the vector of independent variable values, and *b* is the vector of regression coefficients on the logit scale.

The difference between the prevalence estimates of infection in the IGRA- or PPD-tested risk groups obtained through MI and those based only on reported risk categories was expressed as the percentage of bias relative to MI.

LTBI prevalence was calculated by dividing the number of cases in each risk group by the corresponding population size in 2023. The 95% CIs incorporated variability in both the numerator and denominator, assuming three different count data distributions based on the observed right skewness. An exception was made for the state population, which followed a uniform distribution within ±5% of the 2022 census estimate^2^. The selected distributions were log-normal, Poisson, and negative binomial, and the median and interquartile range (IQR) estimates were averaged across the three distributions in a sensitivity analysis of the distributional assumptions. 

To estimate the number of people living with HIV in the state in 2023, a correction factor of 1.59 was applied to account for HIV underreporting between 2008 and 2017. This factor defined the upper bound of the 95% CI^21^, while the SINAN-reported value served as the lower bound of a plausible interval. In the absence of data on variability, the upper bound of the interval was assumed to be 50% above the observed value.

The size of the population of patients with diabetes mellitus treated in the public health system (SUS) in the state in 2023 was estimated using hospital records, assuming that one-quarter of these represented the same individual, treated both as an outpatient and inpatient^19^. The assumed error margins for the SIH and AIH were ±10% and ±30%, respectively, uniformly distributed within the interval. 

The variability in the number of people who use drugs in the state was estimated based on two definitions of crack use from the 3rd National Survey on Drug Use in the three largest cities of Santa Catarina (Florianópolis, Joinville, and Blumenau): use in the past 30 days and use in the past 12 months^16^. This range of estimates was assumed to represent 95% of the log-normal distribution of the number of users.

The variability in the estimated size of the population experiencing homelessness^22^ accounted for potential underreporting of up to 50%.

Estimates of the immigrant population were derived from the Federal Government’s Unified Registry for Social Programs, known in Portuguese as “Cadastro Único,” and compared with external data sources^23^ ^,^ ^24^. The maximum value was estimated as 50% higher than the registered count.

Smoking prevalence among individuals aged ≥18 years in the state of Santa Catarina^18^ was assigned a ±5% margin of error.

Using the mean and standard deviation, 10,000 sampling simulations without replacement were performed for both the numerator and denominator. The 25th, 50th, and 75th percentiles were calculated to derive the median and IQR, which were used to describe the central tendency and typical variation in skewed prevalence distributions.

Statistical analyses were performed using Stata software (StataCorp, 2022)^25^.

### Ethical issues

The study was approved by the Human Research Ethics Committee under the protocol number CAAE 79499224.4.0000.5361. 

## RESULTS

Only 14.3% of laboratory records for IGRA testing in 2023 included information on the risk category (Table 1).

**TABLE 1:** Characteristics of IGRA-tested individuals with known risk category (N = 204).

Characteristic	Category	Statitistic	Risk category						
			Diabetes	HIV/imune supression	Migrants	Institutionalized	Homeless	Smoker	Illicit drugs
**Sex**	Male	n	1	91	6	1	2	5	0
		%	0.94	85.85	5.66	0.94	1.89	4.72	0.00
	Female	n	4	85	1	1	0	6	1
		%	4.08	86.73	1.02	1.02	0.00	6.12	1.02
**Age (years)**	0‒4	n	0	2	0	0	0	0	0
		%	0.00	100.00	0.00	0.00	0.00	0.00	0.00
	5‒14	n	0	3	0	1	0	0	0
		%	0.00	75.00	0.00	25.00	0.00	0.00	0.00
	15‒24	n	0	10	1	0	0	0	1
		%	0.00	83.33	8.33	0.00	0.00	0.00	8.33
	25‒44	n	1	68	5	0	2	3	0
		%	1.27	86.08	6.33	0.00	2.53	3.80	0.00
	45‒64	n	2	68	1	1	0	6	0
		%	2.56	87.18	1.28	1.28	0.00	7.69	0.00
	65+	n	2	25	0	0	0	2	0
		%	6.90	86.21	0.00	0.00	0.00	6.90	0.00
**Race**	White	n	4	153	1	1	2	11	1
		%	2.31	88.44	0.58	0.58	1.16	6.36	0.58
	Asian	n	1	3	1	0	0	0	0
		%	20.00	60.00	20.00	0.00	0.00	0.00	0.00
	Mixed	n	0	14	4	1	0	0	0
		%	0.00	73.68	21.05	5.26	0.00	0.00	0.00
	Black	n	0	4	1	0	0	0	0
		%	0.00	80.00	20.00	0.00	0.00	0.00	0.00
	Unknown	n	0	2	0	0	0	0	0
		%	0.00	100.00	0.00	0.00	0.00	0.00	0.00
**Contact with drug-resistant tuberculosis**	No	n	4	174	7	2	2	11	1
		%	1.99	86.57	3.48	1.00	1.00	5.47	0.50
	Yes	n	1	2	0	0	0	0	0
		%	33.33	66.67	0.00	0.00	0.00	0.00	0.00

IGRA-positive test results revealed that HIV was the main risk factor for LTBI (Table 2). When only the reported risk categories were considered, LTBI prevalence was overestimated by nearly 4-fold among immigrants and 3-fold among tobacco users. Conversely, it was underestimated among PLWHA, immunosuppressed individuals, people who use drugs, those with diabetes, and individuals experiencing homelessness. No LTBI cases were observed among hospitalized individuals, individuals experiencing homelessness, people who use drugs, or individuals with diabetes. Combining observed and MI-estimated data, overall LTBI prevalence among IGRA-tested individuals was 6.89% (95% CI: 5.73-8.05%), 3-4% among immigrants and tobacco users, and 1-2% among other risk groups.

**TABLE 2:** Percentage of IGRA-positive test results based on observed data with reported risk category (N = 204) and missing category (N = 1,222), estimated using multiple imputation. Santa Catarina, Brazil, 2023.

Risk	Observed (reported risk)				MI (unreported risk)			Best estimate*			Relative bias**		
	n	PR (%)	LB (%)	UB (%)	PR (%)	LB (%)	UB (%)	PR (%)	LB (%)	UB (%)	Bias (%)	LB (%)	UB (%)
HIV/immunosuppression	176	9.66	5.29	14.02	19.69	18.48	20.89	18.25	16.59	19.91	-47.08	-51.61	-42.56
Institutionalized, hospitalized	2	0	0.00	84.19	1.97	0.00	5.52	1.69	0.00	16.78	-100.00	-100.00	-96.45
Illicit drug use	1	0	0.00	97.50	1.97	0.00	5.79	1.69	0.00	18.91	-100.00	-100.00	-96.19
Diabetes	5	0	0.00	52.18	1.79	0.00	4.05	1.53	0.00	10.94	-100.00	-100.00	-97.74
Immigrant	7	14.29	0.00	40.21	1.87	0.00	3.80	3.65	0.00	9.01	291.80	265.80	317.79
Homeless	2	0	0.00	84.19	1.99	0.00	5.55	1.71	0.00	16.80	-100.00	-100.00	-96.44
Smoking	11	9.09	0.00	26.08	2.04	0.30	3.79	3.05	0.26	6.98	198.14	181.07	215.22
**Total**	**204**	**9.31**	**5.33**	**13.30**	**6.49**	**5.80**	**7.17**	**6.89**	**5.73**	**8.05**	**35.05**	**31.01**	**39.10**

**Legend:** * Weighted mean of the observed values and MI estimates. ** Observed prevalence with reported risk relative to the best estimate. **HIV:** human immunodeficiency virus. **MI**: multiple imputation; **PR:** prevalence; **LB**: lower bound of the 95% confidence interval; **UB:** upper bound of the interval.

The prevalence of LTBI according to the demographic and epidemiological characteristics of the individuals tested using IGRA is presented in Table 3. Significantly higher percentages were estimated among PLWHA aged under 25 years (24-30%) than among those aged 25 years or older (17-21%). HIV/TB co-infection among self-declared white individuals was nearly 17%, which was significantly lower than that among other ethnic groups (23-33%) and among those reporting contact with drug-resistant tuberculosis (19.28%) compared with the no-contact group (26.81%).

**TABLE 3:** Percentage of IGRA-positive test results, estimated by multiple imputation by risk categories. Santa Catarina, Brazil, 2023.

Variable	Subgroup	Risk Category (%)							
		HIV / Immunosuppression	Institutionalized	Illicit drug use	Diabetes	Immigrant	Homeless	Smoking	Mean
**Sex**	M	18.83	1.87	1.72	1.94	1.74	2.08	2.21	4.34
		(17.09‒20.57)	(0,00‒7.19)	(0.00‒7.72)	(0.00‒5.37)	(0.00‒ 4.52)	(0.00‒7.58)	(0.00‒4.73)	(2.44‒8.24)
	F	20.42	2.05	2.12	1.68	1.99	1.92	1.9	4.58
		(18.76‒22.08)	(0.00‒6.82)	(0.00‒7.04)	(0.00‒4.69)	(0.00‒4.68)	(0.00‒6.61)	(0.00‒4.33)	(2.68‒8.04)
**Age group (years)**	<5	25.31	2.75	1.78	1.96	2.19	1.54	2.69	5.46
		(18.87‒31.75)	(0.00‒0.71)	(0.00‒0.71)	(0.00‒2.21)	(0.00‒0.84)	(0.00‒975)	(0.00‒ 2.71)	(2.69‒9.99)
	5‒14	24.35	2.08	1.91	2.11	1.85	2.34	2.4	5.29
		(20.51‒28.18)	(0.00‒11.4)	(0.00‒0.52)	(0.00‒9.16)	(0.00‒7.75)	0.00‒11.7)	(0.00‒7.96)	(2.93‒1.09)
	15‒24	23.97	1.76	1.35	1.73	1.49	0	1.96	5.38
		(18.23‒29.72)	(0.00‒0.84)	(0.00‒0.84)	(0.00‒11.4)	(0.00‒10.48)	(-.-)	(0.00‒0.55)	(3.04‒1.06)
	25‒44	17.5	2.06	2.1	1.57	1.76	1.97	2.12	4.15
		(15.34‒19.66)	(0.00‒7.53)	(0.00‒9.12)	(0.00‒5.74)	(0.00‒5.23)	(0.00‒7.91)	(0.00‒5.52)	(2.19‒8.67)
	45‒64	18.11	1.64	1.69	1.8	1.75	2.03	1.96	4.14
		(16.17‒20.04)	(0.00‒8.56)	(0.00‒9.00)	(0.00‒5.91)	(0.00‒4.96)	(0.00‒8.55)	(0.00‒4.66)	(2.31‒8.81)
	65+	21.03	2.05	2.25	1.85	2.47	1.74	1.76	4.73
		(17.85‒24.22)	(0.00‒0.46)	(0.00‒10.32)	(0.00‒6.85)	(0.00‒7.84)	(0.00‒10.28)	(0.00‒6.25)	(2.55‒9.46)
**Race**	White	16.83	1.87	1.78	1.63	1.85	1.96	1.95	3.98
		(15.52‒18.15)	(0.00‒5.87)	(0.00‒5.72)	(0.00‒4.17)	(0.00‒4.01)	(0.00‒5.84)	(0.03‒3.87)	(2.22‒6.80)
	Asian	33.27	2.09	2.34	2.75	2.29	2.42	3.24	6.91
		(26.91‒39.62)	(0.00‒0.71)	(0.00‒0.97)	(0.00‒14.09)	(0.00‒0.46)	(0.00‒0.97)	(0.00‒14.2)	(3.84‒1.01)
	Mixed	25.47	1.49	1.9	1.43	1.81	1.92	1.92	5.13
		(21.68‒29.27)	(0.00‒0.60)	(0.00‒0.60)	(0.00‒8.46)	(0.00‒8.14)	(0.00‒0.52)	(0.00‒7.54)	(3.10‒7.88)
	Black	29.92	3.08	9.49	2.94	1.93	2.8	2.89	7.58
		(23.60‒36.23)	(0.00-0.71)	(0.00‒0.71)	(0.00‒13.41)	(0.00‒11.45)	(0.00‒0.71)	(0.00‒12.79)	(3.37‒1.09)
	Unknown	26.89	3.11	0	2.79	1.95	1.53	2.11	6.40
		(20.36‒33.42)	(0.00‒0.52)	(-.-)	(0.00‒14.2)	(0.00‒10.99)	(0.00‒0.97)	(0.00‒11.00)	(3.39‒1.18)
	No	19.28	1.99	1.99	1.81	1.86	2	2.07	4.43
		(18.05‒20.5)	(0.00‒5.58)	(0.00‒5.91)	(0.00‒4.15)	(0.00‒3.82)	(0.00‒5.64)	(0.28-3.86)	(2.62‒7.06)
	Yes	26.81	1.32	1.45	1.49	2.14	1.7	1.58	5.21
		(21.19‒32.43)	(0.00‒0.97)	(0.00‒0.84)	(0.00‒9.89)	(0.00-12.18)	(0.00‒0.84)	(0.00‒9.3)	(3.03‒9.49)

**Legend: F:** Female; **M:** Male.

Table 3 also reveals underreporting among highly vulnerable groups, including children under 5 years of age living with HIV (25.31%), hospitalized individuals (2.75%), people who use drugs (1.78%), tobacco users (2.69%), children of immigrants (2.19%), and children with parents experiencing homelessness (1.54%). An estimated 5.46% of children under 5 years of age lived under such conditions in 2023, a percentage similar to that observed in the 5-14 age group. Among IGRA-tested individuals, nearly 11% were likely children under 15 years of age living in vulnerable settings, and therefore at risk not only for tuberculosis, but also for other diseases.

At the population level, LTBI prevalence per 100,000 inhabitants was estimated at 5.63 (IQR 5.13-6.14) in 2023, with markedly higher rates among individuals treated in the public health system for diabetes mellitus (692), immigrants (588), PLWHA (445), and people experiencing homelessness (352) **(** Table 4 **)**. 

**TABLE 4:** Estimated prevalence of latent tuberculosis infection (LTBI) based on multiple imputation and population size of risk groups in Santa Catarina State, Brazil, 2023.

Risk group	Number of cases	Subpopulation size		Prevalence per 100,000 (IQR)
	(95% CI)	N	Estimated range	
HIV / Immunosuppression	260	47000	47000‒74722 ^a^	897.76
	(237‒284)			(733.52-1099.62)
Hospitalized/Institutionalized	24	25923	25923‒38884 ^b^	92.61
	(0‒239)			(41.45-202.08)
Illicit drug use	24	9000	6000‒9000 ^c^	268.33
	(0‒270)			(49.51-1498.31)
Diabetes mellitus	22	7075	4952‒9197	862.61
	(0‒156)			(258.57-2978.16)
Immigrant	52	82024	82024‒123036 ^b^	596.11
	(0‒128)			(421.83-834.24)
Homeless	24	8824	8824‒13236 ^b^	272.70
	(0‒240)			(121.87-612.60)
Smoking	43	5890985	5596436‒6185534 ^d^	0.03
	(4‒100)			(0.02-0.06)
**Total**	**449**	**7610361**	**7229843‒7990879** ^e^	**5.81**
	**(241‒1417)**			**(1.63-21.72)**

**Legend:** ^a^ Accounting for underreporting^23^. ^b^ Assume a maximum value of 50% higher than that observed. ^c^ Based on two definitions of crack use: in the past 30 days and in the past 12 months^18^. ^d^ Assuming ±5% error (individuals aged ≥18 who smoke ≥20 cigarettes/day)^28^. ^e^ Assuming ±5% error relative to 2022 census data^2^. **CI:** 95% confidence interval; **IQR:** interquartile range.

## DISCUSSION

To the best of our knowledge, this is the first study to estimate the magnitude of LTBI underreporting in Santa Catarina, Brazil, and the epidemiological profile of IGRA-tested individuals using MI to address frequently missing data on risk categories. Based on observed and MI-estimated data **(** Table 2 **)**, the best estimate of tuberculosis infection prevalence among IGRA-tested individuals was 6.89% (95% CI 5.73-8.05%), with the highest rate among PLWHA (18.25%), followed by immigrants and tobacco users (3-4%).

Demographic and epidemiological profiles of cases with missing tuberculosis risk data suggest higher underreporting among PLWHA of African or Asian descent and among those in contact with drug-resistant tuberculosis. Approximately 11% of LTBI cases occur in children and adolescents under 15 years of age. These findings align with reports of 10,628 drug-resistant tuberculosis cases from 2013 to 2022, mostly in males and individuals of mixed race (50.3%). Among the 1,924 new cases, 20.4% had at least one risk factor, including PLWHA (64.9%) and incarcerated individuals (17.3%)^26^. Expanding tuberculosis diagnosis among vulnerable groups is essential for the timely detection and treatment of infected individuals and their contacts, thereby reducing tuberculosis transmission.

Based on IGRA testing and estimated tuberculosis risk group sizes, LTBI prevalence in the general population was almost 6 per 100,000, and between 445 and 692 per 100,000 among immunosuppressed individuals, mainly due to HIV and diabetes mellitus (Table 4). While international studies have reported a high incidence of LTBI among PLWHA, regional data are often lacking. A meta-analysis of 51 studies from sub-Saharan Africa (2000-2022) found a pooled incidence of 3.49 per 100 person-years (95% CI: 2.88-4.17)^27^. The World Health Organization recommends preventive tuberculosis treatment for adults and adolescents living with HIV, regardless of immune status or antiretroviral therapy use, owing to evidence of an additional protective effect^3^. 

In the present study, a high LTBI risk was estimated among people experiencing homelessness who faced precarious living conditions and limited access to basic needs and health services, highlighting the socioeconomic aspects of tuberculosis^5^. The capital of Santa Catarina ranks among the top ten cities with the highest number of people experiencing homelessness in Brazil^14^; therefore, strategies such as directly observed tuberculosis treatment^28^ and Brazil’s *Street Clinic Strategy* could improve access and care quality for this group^29^.

Limitations of this study include uncertainties about the coverage and representativeness of risk groups in the IGRA records, as the data were obtained from a specific laboratory service with unknown referral criteria. Although the Brazilian Ministry of Health recommends IGRA for children aged ≥2 and <10, PLWHA with CD4 counts >350, transplant candidates, and immunosuppressed individuals, IGRA is often applied beyond these criteria^30^. Group size estimates for people who use drugs, people experiencing homelessness, and immigrants typically reflect minimum values and lack clear upper bounds. In such cases, plausible maxima were extrapolated from national data, disregarding regional differences or using wide error margins (e.g., 50%). In this study, the three count data models applied addressed skewness differently, and their precision was limited to the distribution extremes. Although the applied model averaging tended to improve prediction^31^, only future studies with better data can evaluate the extent to which this improvement was achieved. Furthermore, the VIGILANTOS data, by design, do not allow overlap between risk categories, despite evidence to the contrary in various studies on vulnerable groups. Finally, PPD testing for latent TB is still used, but is not reported in the IGRA testing records.

Reducing the uncertainty in the prevalence of LTBI in Brazil requires better data from hard-to-reach populations, which are often underrepresented in medical records. Such data require special sampling schemes, which are typically more costly than those generally applied in epidemiological surveys but may be cost-effective from the perspective of disease prevention.

## CONCLUSIONS

The best estimate of LTBI prevalence among IGRA-tested individuals in Santa Catarina, Brazil, in 2023 was 6.89%, with higher values among PLWHA (18.25%) and immigrants (3.65%). Considerable underreporting was observed among incarcerated individuals, people experiencing homelessness, people who use illicit drugs, and individuals with diabetes, followed by PLWHA, those of African, Indigenous, and Asian descent, and individuals with known contact with drug-resistant tuberculosis. Approximately one-quarter of the IGRA-positive test results were detected among children aged <5 years and slightly fewer among those aged 5‒15 years. The prevalence of LTBI was approximately 6 per 100,000 inhabitants and was highly elevated among those living with diabetes, HIV, immigrants, and people experiencing homelessness.

Collectively, these findings highlight the urgent need to expand targeted testing for LTBI among high-risk groups, with particular attention to children and adolescents aged <15 years, who face a substantially higher risk of progression to active tuberculosis.

## Data Availability

Research data is only available upon request.
